# Factors influencing delivery of intersectoral actions to address infant stunting in Bogotá, Colombia – a mixed methods case study

**DOI:** 10.1186/s12889-020-09057-x

**Published:** 2020-06-13

**Authors:** Natalia Botero-Tovar, Gina Paola Arocha Zuluaga, Andrea Ramírez Varela

**Affiliations:** 1 Population Health Division, Fundación Santa Fe de Bogotá, Bogotá, D.C., Colombia; 2 Universidad de los Andes School of Medicine, Bogotá, D.C., Colombia

**Keywords:** Intersectoral actions, Public-private partnerships, Nutrition, Stunting, Public health

## Abstract

**Background:**

Intersectoral actions (ISA) are a recognized relationship between the health sector and other sectors to improve health outcomes. Although a frequent topic in public health studies, evidence for systematic evaluation of implementation of ISA is scarce. An intersectoral health intervention for infants under one-year-old with, and at risk of, stunting (low height-for-age) was developed by a public-private partnership in Bogotá, Colombia, during 2018 and 2019. Here we report a case study conducted in parallel to the intervention designed to assess factors that influenced implementation of the ISA.

**Methods:**

The case study was developed using a concurrent mixed-methods design, with the qualitative component giving context to the quantitative results. The qualitative component was obtained from four workshops, three focus groups, and 17 semi-structured interviews with actors involved in the intersectoral intervention. The quantitative component was obtained with two questionnaires that evaluated perceptions on improvement and partnership functioning of the ISA.

**Results:**

This study collected information from 122 participants. The intervention demanded intersectoral collaboration. Political will, motivated human resources, and recognition that health improvement results from collaboration, were factors that facilitated intersectoral actions. Intersectoral actions were limited by difficulties in engaging the health sector, communication challenges related to local health service decentralization, and administrative barriers.

**Conclusions:**

Intersectoral actions have recently been discussed in the literature due to challenges in implementation and doubts regarding economic outcomes. The implementation of intersectoral public health interventions can be jeopardized by a lack of coordination and management skills.

## Background

Intersectoral actions (ISA) are recognized relationships between part or parts of the health sector and part or parts of another sector to act on health-related issues in a way that is more efficient and sustainable than could be achieved by the health sector alone [1]. This requires considerable coordination efforts and questions the durability of ISA, what Greer and Lillvis described as “bureaucratic obstacles to intersectoral governance” [2]. For this reason the study of ISA involves detailed attention to plans for monitoring, evaluation, and accountability of the collaborative efforts, with a focus on the importance of soft skills available within the partnership such as communication skills [2–4].

Although ISA is a frequent topic in public health studies, documentation on systematization and implementation is scarce [2, 5–7]. Discussions on the social determinants of health need to unravel the complex forms of intersectoral policy action since health is related to many different institutions and sectors of society [1].

Malnutrition is an example of the call for ISA, and a common topic in governmental agendas that highlights the need for public-private partnerships [8]. Stunting is a form of malnutrition characterized by a low height-for-age that leads to irreversible damage including shorter adult height, lower attained schooling, and reduced adult income [9]. The evidence indicates that stunting is a complex problem demanding action from multiple sectors [7, 10–13]. Global efforts to reduce stunting with an intersectoral approach demand greater attention to communication and articulation of objectives and methods within the different sectors involved in order to accomplish the nutritional goals [7]. A recent systematic review showed that stunting was reduced by multisectoral efforts directed towards community-based nutrition with a strong emphasis on nutrition-sensitive and nutrition specific programs [14]. A recent systematic review by Aguilera and Daher collated macro-level policies and programs for stunting across government, health, and social assistance sectors of several countries including Peru, Malawi, Ethiopia, and Nigeria [14]. Thus, other sectors such as agriculture, social assistance, health, education, water and sanitation, and the private sector have been identified with technical competencies to maximize nutrition results [7]. However, identification of the various sectors’ responsibilities and roles does not necessarily mean that effectiveness of ISA is guaranteed, nor that a favorable environment for collaboration between sectors ensues.

Conditions for ISA include political will, legislative support, health sector engagement, local health service decentralization, motivation of human resources, and social participation [6]. These conditions for ISA has been exemplified in countries like Indonesia, where the National Strategy to Accelerate Stunting Prevention (StraNas Stunting) recognized that coordination across all levels of government was critical to align incentives across national, regional, and local governments [15]. Recognition of health as a collaborative outcome, management approach, teamwork skills and techniques, are all factors that trigger ISA [6]. In this regard, the literature regarding ISA implementation emphasizes that sectors must overcome the challenging task of facilitating meeting points across departments and hierarchical levels [16].

## Theoretical framework and context of this research

Only 19 out of 46 Latin-American and Caribbean countries have reported information on stunting, wasting, and overweight in children under 5 years old between 1985 and 2014 [17]. In 2015, the prevalence of stunting in Colombian children under 5 years old was 13% in the capital city, Bogotá, and 10.8% nationwide [18].

In 2018 and 2019 infants with, and at risk of, stunting were identified in three localities of Bogotá by an intersectoral intervention from government, health, social assistance, and private sectors [18]. In Bogota the provision and administration of health, education and social services are devolved services overseen by the corresponding District Secretariats under the coordination of the Mayor’s office.

The specific aim of the intervention was to promote interaction between the health and social assistance sectors in order to standardize nutritional practices and harmonize knowledge of infant and young child feeding (IYCF) national guidelines across these sectors. It had four main focus points. First, to provide access to good quality health services and the training of health professionals in IYCF practices. Second, to enable access to social assistance programs and training of social assistance professionals in good IYCF practices. Third, to educate caregivers in nutrition practices. Fourth, community empowerment to address stunting. The private sector played a key advocacy role by liaising with stakeholders, providing financial support and access to human resources for implementation of the intervention.

The intervention was developed as a “before and after” study to assess the change in anthropometric measurements of infants identified at risk of stunting or already stunted. The intervention also had an educational component in nutrition for parents and caregivers and was supplemented with food vouchers for those participants. In parallel, this case study was conducted to identify factors that facilitated or limited ISA implementation in Bogotá. The research used concepts extracted from The National Ministry of Health Guidelines for ISA [6]. Definitions are described in a supplementary table (see Annex 1).

## Methods

**Fig. 1:**
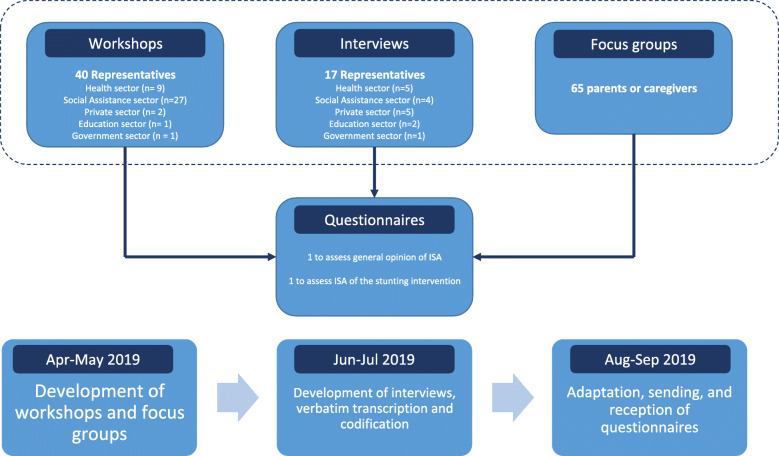
Description of the units of study, data collection instruments, and timeline

The case study was developed using a concurrent mixed-methods design, with the qualitative component giving context to the quantitative results in order to identify factors that facilitated and limited demands, conditions and triggers for ISA in the intervention. We conducted workshops, focus groups, and semi-structured interviews. With the adaptation of two questionnaires, we sought the opinion of the participants to define the best ISA scenario to address stunting and optimize the intersectoral functioning of the partnership. A convenience sample was selected from representatives of the health, social assistance, education, and private sectors. Parents and caregivers also participated. Selection required understanding the purpose of the intervention. A total of 122 individuals participated in this study. Invitations to participate were sent via text message or by phone call. Figure 1 summarizes the units, data collection instruments and timeline of the study.

### Qualitative component

#### Workshops and focus groups

Four participatory workshops were developed. The purpose of the workshops was to assess perceptions regarding demands for ISA on stunting in Bogotá. Participants of these workshops were representatives from multiple sectors (e.g. health, social assistance, education, private), and representatives of local nutrition committees who were familiar with the stunting intervention. Focus groups were developed with parents and caregivers participating in the stunting intervention to identify their perceptions of the reasons for growth faltering, and which sectors they considered most important for infant nutrition. Workshops and focus groups were conducted during April and May 2019.

#### Semi-structured interviews

Interviews were audio-recorded and then transcribed verbatim. Seven committee stakeholders composed of representatives from the health, social assistance, and private sector and three members of the intervention who conducted the fieldwork were interviewed between June and July 2019. Three representatives of health insurance companies agreed to be interviewed to explain the coordination barriers that impeded their participation in the intervention. One representative from the local town hall, and two representatives from the district education sector offered their ideas about how these sectors could contribute to the prevention and treatment of stunting in the localities where the intervention was developed. Guidance for the semi-structured interviews were revised by the second author of this article and an anthropologist. Annex 3 presents the overview of the semi-structured interviews.

Transcribed interviews and fieldwork notes underwent two levels of analysis. The first level used an a priori code list based on the Ministry of Health conceptual guidance for ISA [6], the study protocol, and interview guides. Content was validated by an anthropologist and tested using an initial set of transcripts (Annex 4). NVivo 12 Plus software was used for coding.

The second level of analysis looked at each code individually. Themes, patterns and key quotations were identified. Data saturation was determined as repetition among sources and completion of category definitions. Instruments for data collection also contributed to triangulation, since comparisons were made between participant perceptions of ISA during workshops and interviews. The Consolidated Criteria for Reporting Qualitative Studies (COREQ) checklist was used as a guide to describe important domains concerning the research team, study designs, analysis, and findings [19].

### Quantitative component

#### Questionnaires

Two questionnaires were adapted for quantitative assessments and national experts on ISA conducted content and statistical validation. One questionnaire collected general opinions regarding factors that demand ISA for stunting in Bogotá. Questions were adapted from the stunting conceptual framework of the World Health Organization [20]. Questions to assess general opinions regarding factors that condition and trigger ISA for stunting in Bogotá were adapted from the “Orientations for Intersectoral Actions” [6]. This questionnaire was sent via email in September 2019 to participants of workshops, focus groups and interviewees who had an email address and agreed to receive and answer the questionnaire. The email had a link to collect answers through “Google Forms”. A reminder to answer the questionnaire was sent via email a week after the first mail. Descriptive analysis and calculation of the mean of each answer was performed.

To evaluate perceptions of the functioning of ISA in the intervention against stunting, a second questionnaire was adapted from the Checklist for Intersectoral Partnership for Health Promotion by Mahmood et al. [21]. Thirty-two of the 49 questions of this checklist were adapted. Reasons for adaptation were that the intervention had its own audit for resources, and moreover some of the questions were redundant. The Likert scale was used and a “no answer” option added. The questionnaire was sent via email in September 2019 to members of the intervention committee with a link for collection through “Google Forms.” A reminder email was sent a week afterwards. The mean of each domain was calculated and then a spidergram was graphed to show strengths and weaknesses in the functioning of ISA for the stunting intervention. Full description of questionnaires can be found in Annex 5.

## Results

Forty out of 124 representatives participated in the workshops for a response rate of 32%. Sixty-five out of 90 parents and caregivers participated in the focus groups for a response rate of 72%. Seventeen of 18 of all intended participants in the semi structured interviews attended personally for a response rate of 94%. Thirty-three out of 99 participants of workshops, focus groups and interviewees answered the questionnaire regarding their general opinion of stunting for a response rate of 33%. Nine out of 10 members of the intervention committee answered the questionnaire of ISA for stunting for a response rate of 90%.

### Demands for ISA

Participants of workshops indicated that ISA for stunting must incorporate sectors other than health such as the executive branch of the government, social assistance, education, economic development, environment, the community, and the private sector. Two representatives from the education sector, when asked about their potential role in the prevention of stunting, responded that the district education sector could aid towards reducing teenage pregnancy incidence and improving food security for those already pregnant. They also confirmed that they were aware of *“the consequences or sequelae of inadequate treatment [for stunting], and saw it reflected in some indicators in subsequent years, especially in school dropout*”.

Parents and caregivers highlighted the importance of the health and social assistance sectors for infant nutrition. Notably, some caregivers were less concerned about the stunted child, and more about the child who failed to be ‘chubby’, which was interpreted as a healthy nutritional state.

In one of the interviews the question arose as to why only health and social assistance sectors were the focus of ISA for the intervention. In this regard, a committee member responded: “*we thought about stunting from the conceptual framework of the project as a problem that surpassed just the health sector and we needed to add the social component. For this reason the District Secretariat for Social Integration sent a representative, and, when we were recruiting participants, we approached the Colombian Family Welfare Institute who also sent a representative*”. Another member of the committee explained: “*we started this project with a view on intersectoral actions, since we had to [coordinate between] two Secretariats – social integration and health – and the third actor that was the private sector*”. Although the health, social assistance, and private sectors were invited to participate, some members of the committee felt that additional sectors could have been invited, such as the education sector, economic development, and perhaps the District Secretary for Women. The sectors for the intervention were selected based on their institutional mission statements regarding infant nutrition, those that complied with this requirement were the health and social assistance sectors, and two private institutions.

### Conditions for ISA

As an example of “political will”, a member of the committee highlighted that the Mayor of Bogota authorized the implementation of the stunting intervention through the collaboration of the health and social assistance secretariats. The main role of the health secretariat was to identify infants at risk or with low Height-for-Age Z score (HAZ) registered in their database. The main role of the social assistance sector was to cross-check this information to identify infants affiliated to their programs and include them in the intervention. Helpfully, a member of the committee from the social assistance sector was already working on public-private partnerships, especially on technical support and supervision of legal agreements between the two, which facilitated ISA in the intervention. The private sector assumed a notable advocacy role by promoting the intervention within national and local government entities.

Lack of the following factors limited the ISA, legislative support, full engagement of the health sector, decentralization of local health services, and lack of social participation. In regard to the lack of legislative support for prevention of stunting, a member of the committee from the health sector mentioned that “*unfortunately, in the current plans [of the District Health Secretary] stunting is not an explicit goal. When we talk about prevention of stunting, we talk about breastfeeding, about which we do have a goal.*” The same barrier was highlighted in the workshops, and participants mentioned the lack of legislative support for surveillance of stunting in the city. Wasting or low weight for age (WAZ), is a priority for many sectors. For stunting, as mentioned by the principal investigator, “*there is a normative barrier, [because] we do have an obligation to address wasting [intervention, since] children can die, so it sets alarm bells ringing*” . An interviewee of a health insurance company also stated that they focus their attention on low WAZ.

The lack of full engagement on the part of the health sector was criticized as it did not include key actors, the health insurance companies. The principal investigator commented that “*although we were already represented in the health sector by the District Health Secretary, this sector was not fully represented because we did not have the main actors, the health insurance companies.*” A member of the social assistance sector in the intervention committee highlighted the importance of participation by health insurance companies, and ISA that could not be accomplished by other sectors. In her words: “*without them I think we are incomplete because early identification [of stunting] is not going to happen [ …*], *or if we give one message and they give a different one, we will be in conflict, and also I think that the societal profile of doctors is higher in our culture than that of any other educational agent.*” This interviewee was referring to trained social assistance personnel who give advice in IYCF to parents and caregivers.

Local health service decentralization presented barriers to harmonize knowledge in IYCF. For example, healthcare workers from insurance companies had given IYCF advice to the parents and caregivers which was outdated in view of recent national guidelines, so harmonization was not feasible as these healthcare workers were not trained. Additionally, although indications were given during the intervention, these healthcare workers could not join the intervention due to time constraints.

There was a barrier to “social participation” by some community leaders who were lost to follow up due high migration in and out of the city for personal reasons.

### Triggers for ISA

Regular meetings of the committee were important for coordination efforts. According to a committee member, “*these meetings help us to ask ourselves about how to end or mitigate barriers and work together*”.

Core institutional objectives of the private sector included qualification of the public sector to improve health and social assistance services for infants. A representative from the private institution that funded the intervention highlighted that in the meetings to arrange the partnership, both private and public sectors agreed on the need for a third party to drive the intervention, since “*we are not allowed to give resources to them [public institutions], nor they are allowed to receive these resources [ …*]. *We qualify those institutions through a third party [ …*] *in charge of channeling resources.*” Thus, another private sector institution was designated for this role.

A further trigger for ISA was “recognition of health as a collaborative outcome”. The principal investigator said that “*we accomplished the project in order to study the problem [of stunting], to participate in the intervention, and measure the change, and to present information and policy recommendations that are the final result of this exercise.*” The implementation of the intervention and the elaboration of a guideline for prevention and management of stunting gathered sectors towards a common goal.

The main factors that limited the triggering of ISA were related to “management approach”. Members of the committee highlighted bureaucratic barriers to establish a contractual partnership with health insurance companies. For example, during implementation there were administrative barriers for nurses and medical doctors to receive training during working hours. A health insurance company representative indicated that “*professionals were not allowed to spend eight hours of training on [identification of] stunting*”. Also, “*the project’s expectations could not be accomplished because training [for identification of stunting] was planned during a time of the year when upper respiratory problems are increased and were a priority*”. Virtual training was proposed but the approach was not within the context of the ISA and difficult to implement.

Barriers to information sharing were identified during implementation. Recruitment of infants was hampered by inconsistent updating of data shared between sectors. Each sector had its own database and these were incompatible for data sharing and moreover hindered by data protection laws. According to a committee member, “*intersectoral actions were presented to find infants [at risk or with stunting], but databases were useless to find them”.*

**Fig. 2:**
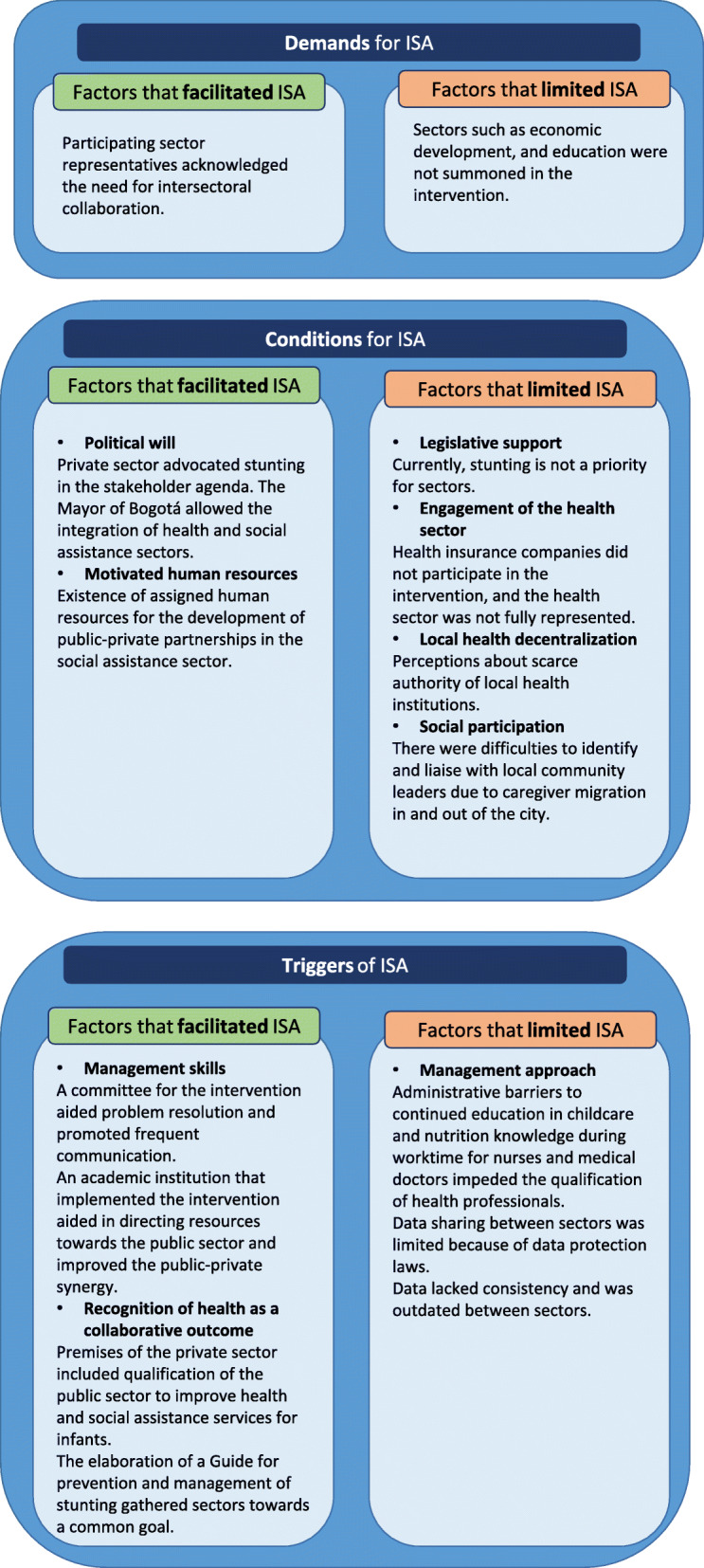
Factors that facilitated and limited ISA grouped by demands, conditions, and triggers for ISA

Figure 2 summarizes factors that facilitated and limited ISA grouped by demands, conditions, and triggers for ISA.

## General opinions on the demands, conditions, and triggers for ISA to address stunting

Factors related to the failure to achieve the potential height-for-age were most frequently ascribed to family income (76%), food quality (76%), and health services (64%). The most frequent conditions for ISA addressing stunting in the three localities were the need for local government coordination between the involved sectors (66.6%), the capacity and authority to guarantee infant nutrition services by the health sector (57.1%), and sufficient resources for the intervention (54.8%). Other factors conditioning ISA for stunting were the need for motivated and IYCF qualified human talent across sectors and an alignment of IYCF guidelines between sectors.

Finally, triggers for ISA were ascribed to the perceived importance of health outcomes by each of the participating sectors (69%), that coordinated and participatory ISA are explicitly promoted (64.3%), and that ISA guarantee interactions between sectors by work group dynamics and/or community participation (59.5%). Results are shown in Table 1.

**Table 1:** Factors that demand, condition, and trigger ISA intervention against stunting in Bogotá

	***N*** = 42	%^**a**^
**Factors that demand ISA for prevention and management of stunting**		
Family income.	32	76,2
Health services.	27	64,3
Educational level of caregivers.	23	54,8
Vulnerable condition of mothers.	19	45,2
Quality of food.	32	76,2
Water and sanitation.	16	38,1
No answer	0	0,0
Others	8	19,0
**Factors that condition ISA for prevention and management of stunting**		
Local governments comprehend the importance of summoning different sectors to solve the problem (Political will).	28	66,7
Support from the current legislation (Legislative support).	18	42,9
Local health institutions have the capacity to solve the problem (Engagement of the health sector)	24	57,1
Sufficient local resources to pose?? the problem and commitment of each sector (Local health decentralization).	23	54,8
People working in the locality are committed to teamwork within representatives of other sectors (Motivated human resources).	23	54,8
The community as a sector is considered the most important for healthy growth of infants (Social participation).	19	45,2
No answer	0	0,0
Others	4	9,5
**Factors that trigger ISA for prevention and management of stunting**		
Interventions that tackle the problem can promote collaborative and participatory interactions between sectors (Management approach).	27	64,3
A leading sector to tackle the problem could be identified (Management skills).	18	42,9
Interventions that tackle the problem guarantee dynamic sector interactions working with the community (Teamwork skills).	25	59,5
Interventions that tackle the problem use technologies and other knowledge management skills (Management techniques).	17	40,5
Interventions that tackle the problem guarantee that each of the participating sectors is concerned about contributing to health results (Recognition of health as a collaborative outcome).	29	69,0
No answer	0	0,0
Other	2	4,8

^a^Total adds up to more than 100% due to multiple choice answers

## Strengths and weaknesses of partnership functioning

**Fig. 3:**
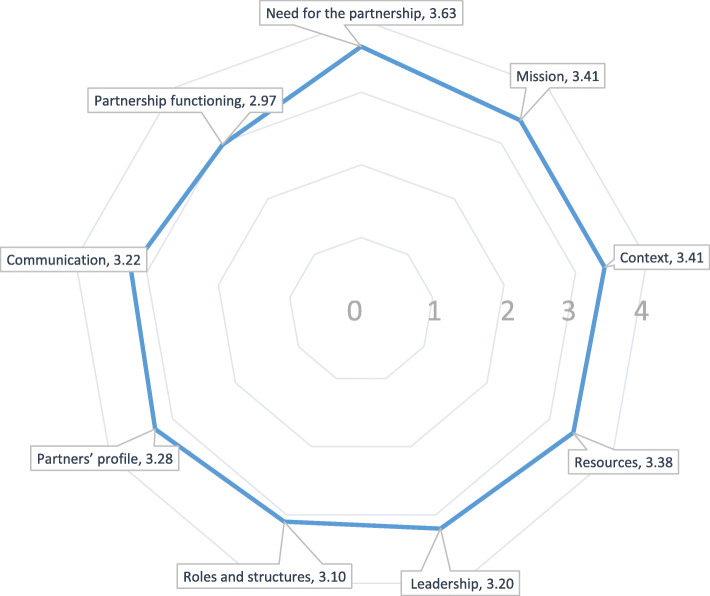
Strengths and weaknesses identified by the members of the intervention committee about partnership functioning. List of concept definitions as adapted from Mahmood et al. [21]. ,*Need for the partnership:* the benefits of a partnership approach for the intervention were clear. *Mission:* refers to the purpose of a partnership for the intervention and encompasses the idea of a shared vision and aligned goals. *Context*: refers to the juncture at which the intervention emerged and within which the partnership existed. *Resources:* financial and other resources such as time, skills, expertise, personal networks and connections for the intervention. *Leadership:* refers to the members of the intervention committee that lead and provided strategic direction to achieve the partnership mission. *Roles and structures:* refers to the level of working agreements within the members of the intervention committee for the partnership. *Partners’ profile:* refers to the overall skills, expertise, and willingness to share resources of members of the intervention to fulfil the mission. *Communication:* the ways in which members of the intervention conveyed information inside and outside of the partnership. *Partnership functioning:* tasks and activities that helped maintain a productive partnership pertaining to the partnership mission

The responses to the adapted “Checklist for Intersectoral Partnerships for Health Promotion” [21] (Annex 5) showed higher agreement between members of the intervention committee in the categories: Need for the partnership, Mission, Context, Resources, Leadership, Roles and structures, and Partners’ profile (Fig. 3). Members of the committee agreed that strengths were:

- they comprehended the need for collaboration between sectors because of common interests and capacity complementarities
- the intervention made use of intersectoral committees already existing in the city
- they considered the possibility that some sectors did not understand the relevance of their own participation
- in the planning and implementation of the intervention, the sectors provided time, human talent and other materials for intersectoral collaboration
- the intervention showed the level of importance that each sector ascribes to health outcomes
- the mutual respect shown by the member sectors.

Categories that showed lower agreement were Communication and Partnership functioning. Members of the committee agreed that weaknesses were:

- the intervention allowed for strategic partnerships to surpass institutional limits
- the roles of each member depended on tasks of other members or sectors
- the partnership allowed for the participation of the community as a sector
- plans to monitor and evaluate the partnership were considered
- plans for problem solving regarding communication and leadership were considered in the partnership

A summary of strengths and weaknesses of the partnership functioning are described in Fig. 3.

## Discussion

To our knowledge, this case study is the first to examine facilitating and limiting factors of ISA in the implementation of a stunting intervention, a prevalent but highly preventable public health problem. The intervention was facilitated by an acknowledgment of the need for intersectoral collaboration, political will, motivated human resources, management skills, and recognition of health as a collaborative outcome. Limiting factors were barriers to the timely summoning of some sectors, a lack of legislative support, insufficient engagement of the health sector, scarce local health decentralization, and barriers for social participation.

Colombia has established ISA as a core tool for development and promotes monitoring and evaluation of ISA to achieve the Sustainable Development Goals (SDG) [22]. There is an emphasis worldwide on documenting the challenges in ‘implementation’ of ISA and their ‘effectiveness’ of outcomes [4, 5, 23]. However, ISA for childhood programs and services have shown there is a need to ensure convergence between sectors and within a variety of programs to attain the common objectives [23]. Thus, prevention and management of stunting demands efficient coordination of ISA to reduce all forms of malnutrition, and to achieve a 40% decline in the number of stunted children under 5 years in Colombia by 2025 [24].

The conceptual framework for stunting by the World Health Organization [20] has previously highlighted that the factors identified demand ISA to address stunting. The context that affects stunting is molded by political economy, health and healthcare services, education, society and culture, agriculture and food systems, and water, sanitation, and environment [20]. In our case study family income was a key underlying factor for stunting. The intervention developed in Bogotá did not cover all of the structural social determinants of stunting. At the juncture of defining sectors related to infant nutrition, those directly involved were the health and social assistance sectors, and private institutions that shared infant nutrition as their mission. However, the literature also supports the importance of including economic factors for optimal linear growth. Children living in poverty lack the appropriate care, stimulation, or nutrition required to promote their development [25].

Research on ISA to address stunting suggests that the government sector should take a leadership role for coordinating ISA [11, 26]. The city of Bogotá has recently delivered a remarkable performance with ISA by achieving an absence of deaths by wasting in children between 0 and 5 years old [27]. This was accomplished by means of ISA strategies described in the literature [28]. The health sector provided therapeutic formula and assistance to prevent child mortality. Legislative support summoned sectors that aided food safety. Intersectoral action in local nutrition committees allow identification and follow-up the cases of child wasting. Social participation strengthened community networks working towards food safety. Notably, the lack of some of these conditioning factors were outstanding barriers for the planning and implementation phases of the stunting intervention. There is a challenge to summon ISA for stunting which does not raise the same degree of awareness as wasting does, especially in the context of declining nutrition-related child mortality [25].

A further conceptual point for discussion regarding differences between intersectoral and multisectoral interventions for stunting arises from the analysis of factors that condition ISA. Nutrition-specific interventions rely on the health sector [7] but there is ample recognition that many additional sectors have roles to play in nutrition-sensitive interventions [11, 29–31]. During planning and implementation of the intervention against stunting in Bogotá, a lack of engagement of the health sector was frequently seen as a barrier for ISA. This may stem from a misconception that health professionals play the pivotal role in infant nutrition. This raises the question of whether the health sector should lead an approach that tackles a nutritional condition rooted in social determinants. Kim et al. have shown that the health sector usually controls the review of indicators thereby limiting the notion of health as a collaborative outcome, in itself a trigger of ISA [4]. More evidence is therefore needed to understand how sectors summoned to address stunting are more effectively interconnected [32].

Recognition of health as a collaborative result triggered ISA in the intervention. Pelletier et al. showcased the difficulties that Peru, Bolivia and Guatemala faced in policy formulation and translation into concrete operational plans [33]. In this regard the World Bank pointed out that sectors should include explicit nutrition indicators in the design phase of interventions [7]. This would help reduce concerns over failure to accomplish sector-specific goals, and remove limits to joint planning and coordination that frequently hinder ISA [4, 12].

Greer and Lillvis argue that obstacles for intersectoral governance are related to coordination and durability, and that solutions lie in enhanced political leadership, bureaucratic change, and indirect strategies such as expanding the range and capacity of people who have access to data, including ordinary citizens [2]. In the context of public-private partnerships of varying and separate organizational regulations, achieving appropriate intersectoral and interprofessional collaboration is complex, especially in private–public engagements [3, 34]. A first step for ISA resides in information quality and sharing among sectors, which is known as informative ISA [6]. Thus, each sector must have clear roles before implementation to avoid barriers and particularly to pre-empt issues with information system management and data sharing [8].

The benefits of ISA to tackle stunting were never questioned by workshop participants and interviewees, nor were the high demands needed for partnerships and synergies. There were however concerns over how to implement ISA. A similar situation was explored in an ethnographic study by Holt et al., who challenged the notion that ISA can be more discursive than practical and thus difficult to implement [5]. Although initiatives for intersectoral or multisectoral strategies to tackle stunting may be approved by the parties concerned, there is a clear need to understand and implement them beyond political will [35].

Lessons learned from this study that may be applicable to other settings are:

- Prevention of stunting demands ISA. A clear message regarding the importance of multiple sector participation to address stunting should be encouraged by researchers and stakeholders.
- Conditions for ISA must be evaluated in the context of the specific intervention.
- Participating sectors must identify common objectives to coordinate activities, and ensure that human and material resources, critical for triggering ISA, are available.
- Factors that influenced delivery of ISA identified in this case study can aid researchers and stakeholders in the design of intersectoral partnerships with robust conceptual frameworks and efficient monitoring of ISA strengths and weaknesses.

This study must be interpreted acknowledging some limitations:

- Recall bias could have been present from the single cross-sectional design of the interviews and the response rate of the questionnaire on general opinion of stunting was low.
- Response rate for workshops was particularly low, but at least one representative of each sector participated. Information saturation of the relevant sectors to address stunting in the city was achieved.
- The adapted Checklist for Intersectoral Partnerships for Health Promotion was applied only at the final stage of the intervention thereby precluding insights from comparison to its application in the early stages of the partnerships and during implementation. The adapted checklist was not completed by members of the intervention committee.

## Conclusions

Prevention of stunting demands ISA at its core, and although the mechanisms to couple actions from the different sectors are well described in the literature, these must be applied within the context of the local actors. This mixed methods case study has highlighted factors related to ISA function, and how stunting was positioned in the government’s agenda and in the mission of the social assistance, health and private sectors in Bogotá, Colombia.

The ISA are highly valued but a lack of coordination and management skills jeopardizes the implementation of the complex interventions needed to integrate the different sectors. Information access and data sharing are pivotal. Constant communication, without losing sight of the demands, conditions, and triggers of ISA, ensure that examination of facilitating factors and the timely resolution of limiting ones results in an effective implementation.

## Supplementary information

**Additional file 1.**

## Data Availability

The dataset, consisting of field notes and interview transcripts, generated and analyzed during the current study are not publicly available to ensure the confidentiality and anonymity of the participating institutions and individuals.

## Abbreviations

ISA: Intersectoral actions
FSFB: Fundación Santa Fe de Bogotá
HAZ: Height for Age Z score
IYCF: Infant and Young Child Feeding
SDG: Sustainable Development Goals
WAZ: Weight for Age Z score
