# Supplementary material for: Factors influencing delivery of intersectoral actions to address infant stunting in Bogotá, Colombia – a mixed methods case study
Source: BMC Public Health. 2020 Jun 13;20:925. doi: 10.1186/s12889-020-09057-x (PMC7293129; doi:10.1186/s12889-020-09057-x)
Supplement: Supplementary file 1 — Additional file 1. [file 12889_2020_9057_MOESM1_ESM.docx]

# Annexes

**Annex 1.** Glossary of terms (6).

| **Term** | **Definition** |
| --- | --- |
| Sector | There are two definition angles. One angle differentiates the public sector from the private sector and the community. This angle relates to public-private partnerships towards improving public services. A second angle considers sectors as different governmental organizations (education, health, agriculture, economic development, culture, housing, etc.). The combination results in government, public, private, and the community sectors (6) |
| Demands for ISA | Events, needs, or health, well-being and quality of life problems with multiple causes that demands an answer beyond the health sector. Implies the planning, organization and integration between different sectors to reach effective resolutions. |
| Conditions for ISA | Existent conditions in the city that allows for intersectoral actions to develop. They are related to society’s capacity to answer to social problems. |
| Engagement of the health sector | Local health institutions strengthened to exercise health authority, and to lead strategies of health for the population. It includes strategies for public health management among different sectors (6). |
| Health decentralization. | The transfer of authority, responsibilities, and resources to local health institutions. Without this, the territory would not be able for leadership, intersectoral actions negotiation, and decision making |
| Legislative support | Current legislation supports intersectoral actions and resources, including explicit objectives for intersectoral collaboration, roles and responsibilities. |
| Motivated human resources | People in the health sector, and other sectors, with full teamwork disposition, and knowledge of intersectoral collaboration. |
| Political will | Full disposition from the governments to assume intersectoral collaboration as a principle to materialize actions for health, wellbeing and quality of life of the population. It is the foundation to summon collective support for a common objective |
| Social participation | Allows for community and citizens to participate and influence in decision making for health policies. It is related with achieving health, well-being and quality of life. It could be used to inform, consult, participate, collaborate, or empower. |
| Triggers of ISA | Management factors that trigger a coherent and coordinated action between sectors and actors in the city. |
| Management approach | A coordinated, strategic, ethical, and participatory approach between sectors. |
| Management skills | Focus and methods of direction. They are related with communication, leadership, motivation, and co-worker’s relationships. |
| Management techniques | Techniques and procedures whose practices have shown improvement in organization implementation like empowerment, direction by objectives or projects, economic health evaluation, benchmarking, outsourcing, the use of technologies of information and communication, and knowledge management. |
| Recognition of health as a collaborative outcome | In health analysis, the social determinants of health are included as key elements to generate social responses, which should consider the programs already existing and the competences of each actor involved. |
| Teamwork skills | Group problem solving and decision making (6). |

**Annex 2.** Description of data analysis categories and coding used.

| **Category** | **Code** | **Pre-established** | **Emergent** | **Instruments** |
| --- | --- | --- | --- | --- |
| Representations of ISA | Definitions of ISA | X |  | Workshops and interviews. |
|  | Sectors for ISA | X |  |  |
|  | Types of ISA |  | X |  |
| Factors related with intersectoral collaboration: that *demand* ISA. | Multiple causes of chronic malnutrition. | X |  | Workshops, interviews, questionnaires. |
| Factors related with intersectoral collaboration: that *condition* ISA. | Political will | X |  | Workshops, interviews, questionnaires. |
|  | Legislative support | X |  |  |
|  | Engagement of the health sector | X |  |  |
|  | Health decentralization. | X |  |  |
|  | Motivated human resources | X |  |  |
|  | Social participation. | X |  |  |
| Factors related with intersectoral collaboration: that *trigger* ISA. | Management approach | X |  | Workshops, interviews, questionnaires. |
|  | Management skills | X |  |  |
|  | Teamwork skills | X |  |  |
|  | Management techniques | X |  |  |
|  | Recognition of health as a collaborative outcome | X |  |  |

**Annex 3.** Overview of the semi-structured interviews

| **Interviews for representatives involved in the intervention**  *Members of the intervention committee*   - 2 Representatives of the district Health Secretary, June 2019. - 2 Representatives of the district Social Inclusion Secretary, June 2019. - 1 Representative of the Colombian Institute of Family Welfare, June 2019. - 1 Representative of Fundación Éxito, July 2019. - 1 Representative of Fundación Santa Fe de Bogotá, June 2019.   *Operative members of the intervention*   - 3 Representatives of Fundación Santa Fe de Bogotá, July 2019. |
| --- |
| **Interviews for representatives of institutions invited to join the intervention**   - 3 Representatives of two health insurances companies, July 2019. |
| **Interviews for people with knowledge of ISA on nutrition or ISA in Bogotá**   - 1 Expert and national adviser on nutrition, July 2019. - 2 Representatives of the district Education Secretary, July 2019. - 1 Advisor of a local town hall, June 2019. |

**Annex 4.** Semi-structured interview guides.

**Members of the intervention committee**

| **Questions** |
| --- |
| Describe your role in the institution that you represent. |
| What generated this project? |
| How were the participating sectors selected? |
| What other sectors could have been part of the project? Why? |
| From your sector, what are the main challenges to position stunting? |
| How would you define intersectoral action? |
| Which are key sectors for intersectoral action for stunting? |
| How do intersectoral actions contribute to stunting prevention or treatment? |
| Do you think that the project achieved intersectoral actions? How? |
| Which achievements of intersectoral collaboration do you consider that the project did manage to obtain, and which ones did not? |
| How did you communicate with each other and what were your team work strategies to accomplish the objectives of the project? |
| What was the most challenging task in the project implementation for intersectoral collaboration? |
| Did you identify opportunities for intersectoral collaboration in this project? Which ones? |
| Did you identify difficulties for intersectoral collaboration in this project? Which ones? |
| How would you describe the community participation in the project? |
| What expectations of the project were accomplished in terms of intersectoral collaboration? |
| What key messages would you give the government for future intersectoral collaboration for stunting in Bogotá? |

**Operative members of the intervention.**

| **Questions** |
| --- |
| What were your expectations for intersectoral actions in the project? |
| How would you define intersectoral action? |
| How would you describe your experience in the coordination with other sectors for the project implementation? |
| What facilitated to work with other sectors? |
| What were the difficulties or barriers to work with other sectors? |
| How did you communicate with the caregivers? |
| According to your fieldwork experience, how can intersectoral actions help prevent or treat stunting? |
| Do you identify challenges for community participation and for community interaction with other sectors? Why? |
| What key messages would you give the government for future intersectoral collaboration for stunting in Bogotá? |

**Representatives of 2 health insurance companies**

| **Questions** |
| --- |
| Describe your role in the institution that you represent. |
| What mobilizes a health insurance company to guarantee adequate health services for infants? Which are some of the normative documents? |
| Any normative document oriented towards infant nutrition? |
| How do you think that stunting could be positioned in the actions of health insurance companies? |
| Do you consider any other sector different from health to articulate towards stunting? |
| How would you define intersectoral action? |
| Do health insurance companies participate in intersectoral spaces? Which ones? |
| How would your sector contribute to the prevention and treatment of stunting? |
| Which institution regulates the health insurance companies in the territory regarding services for infants? |
| How does this health insurance company articulate services with those of the district Health Secretary for infant health and nutrition? |
| How do you collaborate with other sectors in the territory? |
| Where you acquainted with the project of stunting in Bogotá? What do you know about it? |
| What were the barriers and opportunities to participate in the project? Why? |

**Nutrition expert and national advisor.**

| **Questions** |
| --- |
| Describe your role in the institution that you represent |
| What are the main challenges in positioning stunting as a public health problem? |
| What are the key sectors for interventions that address stunting? Do you identify a leading sector to fulfill this task? Which one? |
| What are the roles and responsibilities for the sectors? |
| Do you identify difficulties for collaboration of sectors to prevent stunting? Why? |
| What is the current legislative support for stunting at the national and district level? |
| How visible is stunting in current public health policies? |
| Do you identify a key public health policy for the surveillance of stunting? |
| What barriers have you identified to improve vigilance of stunting in Colombia’s legislation? |
| What key messages would you give the government for future intersectoral collaboration for stunting in Bogotá? |

**Representatives of the district Education Secretary.**

| **Questions** |
| --- |
| Describe your role in the institution that you represent. |
| What do you know about the problem of stunting? |
| Where you acquainted with the project for stunting in Bogota? What do you know about it? |
| What were the barriers and opportunities to participate in the project? Why? |
| The education sector has been identified as a relevant sector for the prevention of stunting, why do you consider that this assertion was made? Do you agree with that? |
| How would the education sector contribute to prevent stunting? Does this sector have actions towards infant nutrition? |
| Do you identify other sectors relevant for the prevention or treatment of stunting? Do you identify a sector that should be the leading sector? |
| How would you define intersectoral action? |
| Do you know any document that gives legislative support to articulation between health and education sectors? |

**Advisor of a local town hall**

| **Questions** |
| --- |
| What are the actions that the local town hall develops for infants in the territory? |
| Are some of these actions directed towards infant nutrition? |
| Is stunting contemplated in the local town hall plan? Why? |
| Do you identify sectors for the management of infant nutrition in your territory? Which ones? |
| Do you identify actions for the local town hall for the management of infant nutrition? |
| How could the local town hall articulate with the health sector to improve actions towards infant nutrition in the territory? |
| How could the local town hall articulate with the social assistance sector to improve actions towards infant nutrition in the territory? |
| How are financial resources of the local town hall directed towards infant nutrition in the territory? |
| Do you identify current spaces or social participation meetings lead by the local town hall to position the problem of stunting in the territory? Why? |

**Annex 5. Questionnaires.**

1. **General opinion about intersectoral interventions for prevention and treatment of stunting in Bogotá.**

The project “Development of a public health intervention for infants under one year old at risk of, and affected with, stunting in Bogota” is a public-private partnership for nutrition. The participant sectors are the government, health, social assistance, and private sectors. You are asked to answer the following questions as you were directly or indirectly involved in the planning or implementation of the project. All information is treated as confidential.

*The objective of the following questionnaire is to assess your perceptions about intersectoral actions for stunting in Bogota.*

Intersectoral actions: A recognized relationship between part or parts of the health sector and part or parts of another sector, that has been formed to take action on an issue or to achieve health outcomes in a way that is more effective, efficient or sustainable than could be achieved by the health sector working alone (OMS, 2010).

1. To me, the problem of infants that are not achieving their height for age are is related to social factors like: (Mark one or more as you consider).
2. Family income.
3. Health services.
4. Educational level of caregivers.
5. Vulnerable condition of mothers.
6. Quality of food.
7. Water and sanitation.
8. Other(s), which one(s)?
9. No answer.
10. To me, the most important conditions to approach the problem of infants that are not achieving their height for age in my territory is/are: (Mark one or more as you consider).
11. That local governments comprehend the importance of summoning different sectors to solve the problem.
12. Support from the current legislation.
13. That the local health institution is in charge of managing the problem and is capable of being the health authority in the territory.
14. Enough local resources to position the problem and a committed responsibility of each sector.
15. That people working in my territory are committed to teamwork with representatives of other sectors.
16. That community as a sector is considered the most important one for healthy growth of infants.
17. Other(s), which one(s)?
18. No answer.
19. To me, the most important factors to give a coherent and coordinated answer between sectors that want to tackle the problem of infants that are not achieving their height for age in my territory is/are: (Mark one or more as you consider).
20. That interventions that tackle the problem can promote collaborative and participatory interactions between sectors.
21. That a leading sector to tackle the problem could be identified.
22. That interventions that tackle the problem guarantee sectors interaction with group dynamics and working with the community.
23. That interventions that tackle the problem use technologies and other knowledge management skills.
24. That interventions that tackle the problem guarantee that each of the participating sectors is concerned about contributing with health results
25. Other(s), which one(s)?
26. No answer
27. **Functioning of intersectoral collaboration in the stunting intervention.**

*The main objective of this questionnaire is to explore the factors that triggered intersectoral actions in the project implementation.*

Please, mark one of the following options according to your perception with each statement:

| 1. Strongly disagree | 1. Disagree | 1. Agree | 1. Strongly agree | 1. No answer. |
| --- | --- | --- | --- | --- |

1. How much do you agree with the following statement: ***the need for intersectoral collaboration as a common interest and capacities complementation was noticed in the project.***
2. How much do you agree with the following statement: ***this project showed that intersectoral collaboration was the best way to approach the problem of stunting.***
3. How much do you agree with the following statement: ***during the project implementation, benefits for my sector from intersectoral collaboration were clear.***

1. How much do you agree with the following statement: ***a common mission for intersectoral action in the territory was defined in the project plan.***
2. How much do you agree with the following statement: ***there were formal agreements (i.e. work plans, written records) for intersectoral collaboration among the members of the project committee.***
3. How much do you agree with the following statement: ***Intersectoral actions were related with my sector’s task for the project implementation.***
4. How much do you agree with the following statement: ***the project was planned to articulate with intersectoral committees of the territory.***
5. How much do you agree with the following statement: ***this project had support from political leaders and stakeholders with capacities for political decision making.***
6. How much do you agree with the following statement: ***members of the project committee considered the need of collaboration between the health sector and social assistance sector to share information.***
7. How much do you agree with the following statement: ***members of the project’s committee considered the possibility that some sectors did not understand their role and relevance for intersectoral collaboration in this project as a factor that could impede partnership success.***
8. How much do you agree with the following statement: ***the commitment among participant sectors to produce the guide of stunting prevention and management as an intersectoral result of collaboration was satisfactory.***
9. How much do you agree with the following statement: ***during the planning and implementation of the project, there was time, human talent, and resources invested from the participating sectors.***
10. How much do you agree with the following statement: ***the leading sector for this project was clearly defined in the planning and implementation of the project.***

* If you answered 3 or 4, please mention the leading sector of the project: __________________.

1. How much do you agree with the following statement: ***the member of the leading sector of this project promoted confidence and respect among the members of the participant sectors.***
2. How much do you agree with the following statement:  ***the member of the leading sector promoted problem solving and interaction among the participant sectors.***
3. How much do you agree with the following statement: ***the leading sector of the project established a space for dialogue and to challenge assumptions of the project.***
4. How much do you agree with the following statement: ***my roles and responsibilities in this project were clearly defined***
5. How much do you agree with the following statement: ***this project highlighted the importance of reaching health results for each of the participant sectors.***
6. How much do you agree with the following statement: ***communication among participant sectors was clear.***
7. How much do you agree with the following statement: ***This project allowed for strategic partnerships to overcome institutional limits.***
8. How much do you agree with the following statement: ***All the potential sectors to participate of this project were summoned.***
9. How much do you agree with the following statement: ***members of the project committee had disposition to share ideas for problem solving, and to improve intersectoral collaboration.***
10. How much do you agree with the following statement: ***members of the project committee were respectful between each other.***
11. How much do you agree with the following statement: ***my roles were dependent on tasks of other participant sectors of the project.***
12. How much do you agree with the following statement: ***members of the project committee had opportunities for face-to-face communication.***
13. How much do you agree with the following statement: ***members of the project committee had clear tasks to communicate within their sector.***
14. How much do you agree with the following statement: ***there were efficient mechanisms to share information between participant sectors.***
15. How much do you agree with the following statement: ***decision making was participatory in the project implementation.***
16. How much do you agree with the following statement: ***members of the project committee were aware that the project added value (rather than duplicating services) for the community or institutions involved in the partnership.***
17. How much do you agree with the following statement:  ***in the project planning and implementation, the partnership assured the participation of the community as a sector for decision making.***
18. How much do you agree with the following statement: ***plans to evaluate and monitor partnerships were considered.***
19. How much do you agree with the following statement: ***plans for problem solving regarding communication and leadership were considered in the partnership.***
